# Social Connectedness in Older Adults: The Potential of Social Internet Use to Maintain a Strong and Stable Personal Network

**DOI:** 10.1093/geronb/gbaf014

**Published:** 2025-01-25

**Authors:** Jeroen H M Janssen, Theo G van Tilburg, Erik J van Ingen, Rense Corten, G M E E (Geeske) Peeters

**Affiliations:** Department of Geriatric Medicine, Radboud university medical center, Nijmegen, The Netherlands; Department of Developmental Psychology, Tilburg University, Tilburg, The Netherlands; Department of Sociology, Vrije Universiteit Amsterdam, Amsterdam, The Netherlands; Department of Sociology, Vrije Universiteit Amsterdam, Amsterdam, The Netherlands; Department of Sociology/ICS, Utrecht University, Utrecht, The Netherlands; Department of Geriatric Medicine, Radboud university medical center, Nijmegen, The Netherlands; Radboudumc Alzheimer Centre, Radboud university medical center, Nijmegen, The Netherlands; (Social Sciences Section)

**Keywords:** Digital technology, Loneliness, Social media, Social support, Strong ties

## Abstract

**Objectives:**

Maintaining a strong social network in later life can be challenging due to limited resources, life events, and changes in health. Social internet use provides an accessible way for communication that is less susceptible to age-related challenges. Although social internet use is increasingly used by older adults, we do not know how social internet use shapes older adults’ offline networks. The purpose of this study is to examine whether social internet use can help maintain strong social relationships.

**Methods:**

We used data from 3 waves (2012–2013, 2015–2016, and 2018–2019) of the Longitudinal Aging Study Amsterdam. Our sample included 2,266 older adults aged 55–99 (mean = 68.2 years, 54% female). We included the frequency of social internet use and computed personal network size, contact frequency, and the number of continued, gained, and lost ties over time. Hybrid models were applied to disentangle between within- and between-person associations.

**Results:**

More frequent social internet users had significantly larger personal networks and, relative to the previous wave, more continued and gained network ties, compared to less frequent social internet users. A within-person increase in social internet use over time was associated with more continued and gained ties.

**Discussion:**

Social internet use may help maintain a strong and stable network, which is important for social connectedness in later life. It allows for additional interaction opportunities, as well as network maintenance and growth. Social internet use thus proves to be a valuable addition to the social interaction resources of older adults.

A strong, stable, and large enough social network in later life is important as a source of instrumental, emotional, and informational support that older adults can draw on during difficult life transitions (Broese van Groenou & van Tilburg, 1997; Cohen, 2004; Cornwell & Laumann, 2015). More support is given by larger networks with frequent contact (Ashida & Heaney, 2008), and older adults prefer to receive support from strong ties, as their availability is generally less circumstantial than that of weak ties (Broese van Groenou & van Tilburg, 1997; Kahn & Antonucci, 1980).

However, maintaining this network of strong ties asks that older adults invest time and energy in frequent interactions, as close relationships require sustained reciprocal exchanges (Krackhardt, 2003; Sorkin et al., 2009). At the same time, the energy available to engage in these interactions decreases in later life, due to declines in physical, cognitive, and sensory capabilities (Huxhold et al., 2013; Moeyersons et al., 2022; Smith, 2012). To be able to support older adults in relationship maintenance despite these challenges, alternative ways of communication are needed that are less energy-consuming.

This article focuses on one of such ways, social internet use (SIU), which offers interpersonal communication that requires less energy than face-to-face interactions. SIU has the potential to overcome geographical distance and allow contact with a larger network (Cohen-Mansfield et al., 2016; Newman et al., 2019). Therefore, the purpose of this study is to investigate whether SIU enables older adults to maintain a strong and stable social network.

## The Differential Investment in Resources Model

The notion that social relationships require sustained resource investment is the central tenet of the *differential investment in resources (DIRe) model* of Huxhold et al. (2022). The DIRe model states that the amount of investment of resources determines the number of ties and the structure of the network, as well as the closeness with network ties. It proposes two relevant resources, time and energy. Given that the availability of energy decreases in later life more than the availability of time, we focus primarily on energy.

The DIRe model also proposes different pathways through which individual characteristics influence this investment in social ties. The pathway most relevant for this study is the mechanism that individual differences in health determine the amount of resources available. Specifically, declines in physical or cognitive health in later life greatly decrease how much energy an individual has to maintain or gain social ties (Huxhold et al., 2013; Moeyersons et al., 2022). This means that, over time, older adults will have increasing difficulty to maintain a strong network. This is especially detrimental for geographically distant ties (Cohen-Mansfield et al., 2016), which require a relatively higher energy investment.

To maintain a strong network in later life, alternative ways of communication are needed that can cope with the limited energy availability. One of these ways is SIU. The value of SIU lies in that it is an easy and quick way to communicate, irrespective of mobility or geographical distance. In other words, SIU is assumed to require less energy than face-to-face communication. According to the mechanisms proposed by the DIRe model, this would mean facilitating investments, which would benefit the network (Huxhold et al., 2022).

## Social Internet Use in Older Adults

SIU is defined as the use of online resources that allow individuals to connect with others for social interaction (Nowland et al., 2018). SIU is increasingly adopted by older adults. In 2019, 61% of Dutch adults aged 65 and older used the internet to communicate with others, compared to 29% in 2014 (Statistics Netherlands, 2019).

Although SIU increases in older adults, research has insufficiently explored the mechanisms through which SIU affects older adults’ network structure (Hülür & Macdonald, 2020). In a longitudinal study of individuals aged 15–45, Vriens and van Ingen (2018) found that more frequent SIU was associated with a larger network, a higher contact frequency with alters, and more continued, gained, and lost ties between two measurements. In other words, the network was more dynamic, whereas the result that there was a larger network indicates that the number of continuing and gained ties were greater than the number of lost ties. However, the results by Vriens and van Ingen (2018) cannot be readily generalized to older adults. Given the lower availability of energy to engage in (energy-consuming) face-to-face interactions (Huxhold et al., 2022), it could be that older adults use and benefit more from SIU to maintain a strong network, as in-person communication is more difficult.

This study aims to explore the potential of SIU in affecting the personal network of older adults over time, using the characteristics defined by Vriens and van Ingen (2018). Specifically, we will look at the relationship between SIU and structural characteristics of the network, that is, contact frequency, network stability (i.e., new, continued, and lost ties), and network size.

## How SIU Affects the Personal Network Structure of Older Adults

### SIU and Contact Frequency

SIU offers an additional means of communicating with network contacts (Cotten et al., 2022). Empirical studies using cross-sectional data have shown that SIU increases contact frequency with strong-tie family and friends in a sample aged 50 and over (Hogeboom et al., 2010), as well as with peripheral ties by Kim and Fingerman (2022), who analyzed daily SIU and contact patterns in a sample aged 65 and over. Two previous longitudinal studies found that SIU increased social contact, although they did not distinguish between strong and weak ties (Yu et al., 2021; Zhang et al., 2020). Therefore, regarding contact frequency, we hypothesize:

H1. The frequency of SIU is positively associated with contact frequency with network ties in older adults.

### SIU and Network Growth and Stability

SIU can facilitate network growth and stability. Due to the ease with which people can be found and reached online, new relationships can be forged and maintained. For instance, SIU allows reconnecting with previously dormant ties (Quinn, 2013). In a sample of U.S. adults aged 65+ years, almost 60% used SIU to reconnect with an alter in the year preceding the assessment (Sumner et al., 2021). Moreover, Cotten et al. (2013) found that more frequent SIU was associated with increased agreement that SIU facilitates meeting other people and expanding one’s network. Regarding network additions, we hypothesize:

H2. The frequency of SIU is positively associated with the number of gained network ties in older adults.

SIU can help keep in touch with existing contacts, which is one of the main reasons older adults engage in SIU (Newman et al., 2019). A study on WhatsApp use showed that older adults carefully choose the message content based on their relationship with the receiver and that these exchanges heightened the feeling of relationship closeness (Braunwalder et al., 2021). Thus, it facilitates the reciprocal exchange of support needed to maintain the relationship. We hypothesize:

H3. The frequency of SIU is positively associated with the number of continued network ties in older adults.

Additionally, SIU can help prevent losing ties by making it easier to maintain network ties and to stay in touch with geographically distant contacts (Gil-Clavel et al., 2022). In-person interactions with distant contacts are more difficult, making them vulnerable to being lost. In such cases, SIU, due to lower resource requirements, can prevent these losses. As argued by Ivan and Fernández-Ardèvol (2017), older adults are motivated to use alternative means of communication—such as SIU—in cases where in-person interactions are not possible. Evidence for this comes from qualitative research by Quan-Haase et al. (2017), who interviewed Canadian adults aged 65+ on their SIU behavior and found that participants regarded SIU as a valuable way to maintain long-distance contacts. We therefore hypothesize that more SIU allows more communication opportunities with relationships at risk of being lost, and thus:

H4. The frequency of SIU is negatively associated with the number of lost network ties in older adults.

### SIU and Network Size

We expect that more SIU is related to an increase in continued and gained ties and a decrease in lost ties associated. The net result of this is a relatively larger personal network. In other words, SIU could help mitigate the shrinkage of the network associated with aging (Cornwell et al., 2008) by allowing older adults to maintain a large network (Yu et al., 2016). Although longitudinal studies specifically looking at network size are limited, Kim et al. (2022) found that, following an Internet use promotion program, moderate and frequent SIU users reported larger networks than non-users. We therefore hypothesize:

H5. The frequency of SIU is positively associated with network size in older adults.

## Method

### Sample and Design

We used data from the Longitudinal Aging Study Amsterdam (LASA), an ongoing longitudinal cohort study on physical, social, cognitive, and emotional aspects of aging in Dutch older adults. The medical ethics committee of the VU University Medical Center approved the LASA study. Data were collected through face-to-face computer-assisted interviews. All participants provided written informed consent prior to data collection.

LASA started with a sample of adults aged 55–84 in 1992–1993 taken from the registers of nine municipalities in three regions in the Netherlands, selected to optimally represent the variation in the urbanity and religion of the older Dutch population (Cohort 1, *n* = 3,107). Subsequently, two refresher samples were added in 2002–2003 (Cohort 2, *n* = 1,002) and 2012–2013 (Cohort 3, *n* = 1,023). Data collection repeats every 3–4 years. For further details on design and data collection procedures, see Hoogendijk et al. (2020). We used data from the follow-up observation in 2012–2013 in Cohorts 1 and 2 and the baseline observation of Cohort 3 (T1, *n* = 2,545), and follow-up observations in 2015–2016 (T2, *n* = 2,024), and 2018–2019 (T3, *n* = 1,701).

From the initial 2,545 respondents at T1, we excluded 279 respondents who had missing personal network data for all three waves. Of the remaining 2,266 respondents, 2,222 had valid SIU and network data at T1, 1,663 at T2, and 1,335 at T3. Compared with the respondents who did not participate at T1, the people in our sample were younger, slightly higher educated, and had a larger network. To analyze contact frequency in network relationships, we used a data set structured on the relationship level, containing data of 58,928 network members (i.e., alters) identified in one or more observations.

### Measures

#### Social internet use

Participants answered multiple questions regarding their internet use. First, they were asked, (1) ‘Do you use the internet?’. If answered *yes*, they received the Question (2), “Which of the following internet functions have you used in the past year?” with several options, one of which was (2f), “Maintaining contact with family, friends, or neighbors (e.g., through e-mail, chatting, Skype, or WhatsApp).” Participants who selected Option (2f) received the follow-up question, (3) “How often do you use the internet for contact with family, friends, or neighbors?’ We recoded the response options from an ordinal scale to a scale reflecting the number of days of SIU per year, ranging from 0 to 365 to approach the ratio measurement level. The response options were 3 (*less than a few times per year*), 10 (*a few times per year*), 60 (*a few times per month*), 156 (*a few times per week*), 365 (*daily*). Additionally, we assigned 0 (*never*) to respondents who answered “no” to Question (1) or did not select Option (2f) on Question (2).

#### Personal network

To identify the respondent's personal network, LASA used the domain-contact approach (van Tilburg, 1998). This approach utilizes name-generator questions to identify network members across seven domains: household members (including the spouse, if present), children (including stepchildren) and their partners, other relatives, neighbors, colleagues (including voluntary work or school), fellow members of organizations (e.g., athletic clubs, church, political parties), and others (e.g., friends and acquaintances). The personal network derived from the domain-contact approach comprises the ties within which there is frequent contact and which are most important to the respondent.

For each domain, respondents were asked to identify individuals with whom they had frequent contact and who were important to them. Everyone could only be named once. The measurement design was the same across waves. Interviewers matched the named alters across waves based on their names, sex, and reported relationship type. Matching was subsequently verified regardless of the relationship type to identify alters for whom the reported relationship type had changed.

We derived five outcome variables from the personal network. *Network size* is the number of valid name-generator responses at the respondent level. *Contact frequency* indicates at the dyad level the response to the question, “How often are you in touch with ...?” with response categories recoded to reflect the number of contact days per year: 0 (*never*), 1(*yearly or less often*), 3 (*a few times per year*), 12 (*monthly*), 24 (*once every two weeks*), 52 (*weekly*), 156 (*a few times per week*), and 365 (*daily or household member*). If an alter is not mentioned in a specific wave but is mentioned in another wave, we set the contact frequency to 0 (*never*).

The number of continued, gained, and lost ties are respondent-level variables, created by restructuring the respondent-level data set with three waves to a data set with two sets of subsequent measurements, T1–T2 and T2–T3. For both sets, we indicated whether a tie was mentioned in either measurement T and T + 1 and summed those per respondent. *Continued ties* indicate the number of ties mentioned in both measurements T and T + 1. *Gained ties* indicate the number of ties that are not mentioned in T but are mentioned in T + 1. *Lostties* indicate the number of ties mentioned in T and not mentioned in T + 1.

#### Control variables

We controlled for gender (male/female), age in years, educational level in years, partner status (no partner, co-residing partner, or partner outside the household), and self-reported general health status (ranging from 1 = *excellent* to 5 = *poor*). We also controlled for the combined hours per week spent on paid or voluntary work, as people often have social interactions at work, possibly reducing the need for SIU. More work hours decrease available leisure time that can be spent on SIU (Dal Cin et al., 2023). Furthermore, in the analyses on contact frequency and network stability, we control for baseline network size. Network size is negatively associated with contact frequency (van den Berg et al., 2009), and the number of possible continued, gained, or lost ties depends on the total number of ties in the baseline network.

### Analytical Strategy

We used hybrid regression models to test our hypotheses (Allison, 2009). These models partition the time-varying variables into within- and between-subject effects and simultaneously estimate those in a random effects model. This approach yields both longitudinal (within-person) and cross-sectional (between-person) estimates while controlling the fixed effects for measured and unmeasured time-invariant heterogeneity (Firebaugh et al., 2013).

Hybrid models can thus disentangle predictions about differences between and changes within respondents. This differentiates them from the widely used mixed effects (or multilevel) models. In mixed effect models, the between-subject and within-subject effects are estimated as one single effect, yielding a weighted average of the between- and within-subject part, therefore not allowing to disentangle these effects (Twisk & de Vente, 2019).

We analyzed the effect of SIU on contact frequency (H1) using linear hybrid regression on the relationship-level data set, with cluster-corrected standard errors to account for the clustering of network members in respondents (Rogers, 1993). For the effect of SIU on network stability (H2, H3, H4), we used the data set restructured to two sets of subsequent measurements and applied ordinary linear regression, with standard errors corrected for having two intervals per respondent. For the time-variant predictors, two variables were created: a baseline variable, indicating values at T1 and T2 for the two intervals, respectively, and a change variable, indicating difference scores T2 − T1 and T3 − T2. The effect of SIU on network size (H5) is analyzed using linear hybrid regression on the respondent-level data set.

#### Robustness checks

Network size and the number of continued, gained, and lost ties are count variables, which often do not follow a normal distribution. Poisson or, in the case of overdispersion, negative binomial models account for this. We analyzed the data using Poisson and negative binomial models, but they did not result in conclusions different from those of the linear model. As contact frequency is an ordinal variable recoded as a ratio variable, we also fitted an ordinal hybrid regression model on the dyad-level data, which did not change the conclusions. See Supplementary Tables 1–5 for the results of the various robustness checks.

## Results

### Sample Description

Of the *N* = 2,266 respondents at baseline, 54% were female, and the mean age was 68.2 years (standard deviation [*SD*] = 9.2). Regarding partner status, 69% had a co-residing partner, 27% had no partner, and 4% had a partner outside their household. The mean years of education attained was 10.9 (*SD* = 3.5), and the mean weekly combined paid and voluntary work hours was 10.9 (*SD* = 16.3). The average self-reported health was 2.3 (*SD* = 0.9), indicating good to fair health status. Table 1 shows the sample characteristics for all independent and dependent variables. Respondents engaged in more frequent SIU over time. At T1, 35% reported no SIU and 17% reported daily use, which changed at T3 to 22% and 33%, respectively.

**Table 1.** Summary Statistics of Social Internet Use and Personal Network Characteristics

Variable	Range	*n*	*M*	*SD*
Social internet use	0–365			
T1		2,246	111.8	129.0
T2		1,752	136.7	142.0
T3		1,387	168.4	148.9
Network size	0–80			
T1		2,224	18.4	11.3
T2		1,663	18.1	10.9
T3		1,336	17.1	10.0
Contact frequency	0–365			
T1		49,325	67.7	103.2
T2		49,148	48.2	90.8
T3		41,594	43.0	87.1
Number of continued ties	0–50			
T1–T2		1,614	11.4	7.2
T2–T3		1,296	11.1	6.6
Number of gained ties	0–35			
T1–T2		1,614	3.5	5.1
T2–T3		1,296	6.2	5.5
Number of lost ties	0–57			
T1–T2		1,614	8.1	6.8
T2–T3		1,296	7.6	6.4
Change in social internet use	–365 to 365			
T1–T2		1,734	19.4	132.4
T2–T3		1,371	19.4	139.3

*Notes*: *M* = mean; *SD* = standard deviation.

### Contact Frequency

Results regarding H1 about contact frequency (Table 2) showed nonsignificant cross-sectional and longitudinal associations between SIU and contact frequency. This means that more frequent SIU is not associated with more frequent contact, and an increase in SIU is not associated with an increase in contact frequency. Thus, H1 was not supported.

**Table 2.** Cluster-Corrected Hybrid Linear Regression of Contact Frequency (Range 0–365) on Social Internet Use (*n* = 2,264)

Variable	*b*	*SE*
**Cross-sectional associations**		
Social internet use	–0.003	0.006
Age	–0.702***	0.096
Network size	–1.031***	0.066
Hours of work	0.520***	0.065
Health	3.724***	0.803
Co-residing partner^a^	5.809***	1.703
Partner outside household^a^	–3.432	3.827
**Longitudinal associations**		
Social internet use	0.003	0.003
Age	–1.411***	0.114
Network size	0.867***	0.060
Hours of work	0.475***	0.067
Health	1.336*	0.655
Co-residing partner^a^	0.292	2.090
Partner outside household^a^	0.651	2.921
**Time-invariant associations**		
Female	–0.177	1.341
Years of education attained	–2.058***	0.192

*Notes*: *SE* = standard error. 138,315 observations of 58,852 relationships clustered in 2,264 respondents.

^a^Reference category: no partner.

* *p* < .05.

** *p* < .01.

*** *p* < .001.

### Network Growth and Stability

The analyses for H2, H3, and H4 on network stability (Table 3) show that cross-sectionally, more SIU is associated with more gained and continued ties. On average, compared to older adults without SIU, older adults with SIU “a few times per week” have an estimated 0.9 more gained and 0.4 more continued ties. Longitudinally, an increase in SIU is significantly associated with an increase in gained and continued ties. If a person increases their SIU from never to a few times per week, their gained and continued ties are expected to increase by 0.5 and 0.4, respectively. These findings support H2 and H3.

**Table 3.** Cluster-Corrected Linear Regression of the Number of Continued, Gained, and Lost Ties on Social Internet Use (*n* = 1,624)

Variable	Continued ties		Gained ties		Lost ties	
	*b*	*SE*	*b*	*SE*	*b*	*SE*
Baseline social internet use	0.002**	0.001	0.006***	0.001	0.001	0.001
Change in social internet use	0.003***	0.001	0.003***	0.001	–0.001	0.001
Baseline age	–0.030*	0.014	0.188***	0.014	0.018	0.011
Change in age	0.350*	0.151	1.741***	0.233	0.105	0.229
Baseline health	–0.270*	0.128	–0.217*	0.109	0.147	0.094
Change in health	–0.110	0.106	0.036	0.131	0.048	0.124
Network size at T1	0.429***	0.015	0.088***	0.010	0.394***	0.010
Hours of work at T1	–0.009	0.008	0.009	0.006	0.008	0.006
Co-residing partner at T1^a^	1.700***	0.255	0.394	0.217	–0.818***	0.180
Partner outside household at T1^a^	0.284	0.433	0.185	0.426	–0.225	0.339
Female	1.002***	0.221	0.061	0.198	–0.585***	0.160
Years of education attained	0.069*	0.030	0.068*	0.029	0.021	0.023

*Notes*: *SE* = standard error. 2,884 observations clustered in 1,624 respondents. In these analyses, the three waves have been restructured into two sets of two subsequent waves. “Baseline” has the values of T1 and T2 for the two waves, respectively, whereas “Change in” has the values T2–T1 and T3–T2, respectively.

^a^Reference category: no partner.

* *p* < .05.

** *p* < .01.

*** *p* < .001.

No significant cross-sectional and longitudinal effects were found regarding lost ties, meaning that (a change in) SIU was not associated with (a change in) the number of lost ties in our sample. We found no support for H4.

### Network Size

The results for personal network size (Table 4) showed a significant cross-sectional but not longitudinal association. More frequent SIU is associated with a larger personal network: Older adults with SIU a few times per week have an estimated network of 2.5 ties larger than older adults without SIU. A change in SIU over time was not associated with a change in network size. These results partly support H5.

**Table 4.** Cluster-Corrected Hybrid Linear Regression of Network Size (Range 0–80) on Social Internet Use (*n* = 2,264)

Variable	*b*	*SE*
**Cross-sectional associations**		
Social internet use	0.016***	0.002
Age	–0.048	0.027
Hours of work	0.042**	0.017
Health	–1.116***	0.239
Co-residing partner^a^	4.856***	0.485
Partner outside household^a^	0.557	1.125
**Longitudinal associations**		
Social internet use	0.002	0.001
Age	–0.399***	0.040
Network size	0.009	0.015
Hours of work	–0.202	0.204
Health	0.570	0.662
Co-residing partner^a^	–0.406	0.831
Partner outside household^a^	0.002	0.001
**Time-invariant associations**		
Female	3.855***	0.412
Years of education attained	0.149*	0.060

*Notes*: *SE* = standard error. 5,167 observations clustered in 2,264 respondents.

^a^Reference category: no partner.

* *p* < .05.

** *p* < .01.

*** *p* < .001.

## Discussion

Later life is characterized by a general decrease in the availability of energy needed to maintain social relationships (Huxhold et al., 2022). In this study, we found evidence that SIU has the potential to partially compensate for the loss of resources, by providing a means of communication that required less energy. Using panel data from older Dutch adults, we showed that older adults with more frequent SIU generally have more gained and continued ties and a larger network in general. An increase in SIU over time is associated with more gained and continued ties. These results suggest that SIU facilitates older adults to remain socially active, thereby maintaining or expanding their network.

Contrary to H1, we found no evidence of an association between SIU and contact frequency. This contradicts previous literature suggesting that SIU provides additional interaction opportunities to in-person interaction (Cotten et al., 2013; Kim & Shen, 2020). It is possible that in this sample, older adults increasingly replace in-person contact with contact through SIU, resulting in a net equal contact frequency. This is in line with the DIRe model, suggesting that, given decreasing energy availability and limited mobility, a shift to more contact through SIU might be preferred or even necessary for older adults. However, the alter-level contact frequency data in LASA does not differentiate between online and in-person contact, so this alternative explanation cannot be tested. Future studies are needed to examine how often older adults are in contact with network members through in-person meetings or via SIU, and see whether, with age, in-person contact decreases, while SIU contact increases.

In accordance with H2 and H3, our findings demonstrate that more frequent SIU is associated with having more continued and gained ties. This suggests that SIU may facilitate the continuation of relationships, which is consistent with previous literature (Ivan & Fernández-Ardèvol, 2017; Newman et al., 2019). SIU can be a valuable means of maintaining contact despite the limited resources available in later life to sustain relationships. For instance, reduced mobility and increased distance may impede regular in-person social interaction, which SIU can partially mitigate. A next step could entail investigating whether the ties established and maintained by SIU are predominantly with friends, family, or acquaintances, to better understand the precise role of SIU in preventing network shrinkage.

Contrary to H4, we did not find a significant association between SIU and the number of lost contacts. Although SIU allows for maintaining contact despite limitations such as geographical distance or mobility issues (Gil-Clavel et al., 2022; Quan-Haase et al., 2017), contact through SIU might not be of sufficient quality to prevent losses in the strong-tie personal network. This has previously been described by Cotten et al. (2013), who indicated that although SIU enables older adults to stay in touch with a large network, it does not affect the quality of their interactions. Strong ties are defined by invested time, emotional intensity, intimacy, and reciprocity (Granovetter, 1973), aspects of qualitatively high contact that might be more difficult to achieve through SIU. This suggests that a certain proportion of contact should be composed of in-person contact to maintain strong ties in the network. The question remaining for future studies is what proportion of contact needs to be in-person to prevent tie loss.

Regarding H5 on network size, the significant cross-sectional associations with SIU are consistent with previous literature stating that SIU improves social connectedness (Stockwell et al., 2020). However, we did not find a longitudinal association with network size. One explanation for this nonsignificant finding could be the reversed effect masking the effect of SIU on network size. Older adults may increase their SIU because they experience a deficit in their contacts (Nowland et al., 2018) or to maintain an expanding network. Alternatively, it is possible that SIU cannot fully compensate for the network losses that older adults experience over time. Increasing their SIU, on the one hand, leads to more gained and continued ties, but combined with possible network losses during this period might result in an unchanging net network size.

Many previous studies have focused primarily on using social media such as *Facebook* (Chang et al., 2015; Cotten et al., 2022; Zhang et al., 2020). By using a broader survey question, we also included, for example, online messaging or e-mail. This is advantageous because social interactions also occur through these channels. However, to fully understand the potential of SIU, future research should distinguish between different online communication channels (Cotten et al., 2022). For example, it can show whether network additions are due to membership in online communities that lead to in-person interactions or primarily caused by one-on-one online messaging. Knowing which type of online communication has which benefits can help tailor new interventions to address social connectedness and loneliness on a personal level, providing the optimal tool for a specific personal situation.

The results of this study have several implications. First, they only partly align with Vriens and van Ingen (2018), who found a pattern of network change and instability due to SIU in a sample aged 15–45 years. However, in our study, SIU of older adults is associated with a pattern of stability, facilitating network growth and maintenance. As many older adults still believe that SIU is a nonmeaningful way to spend time, distracting from the goals of nurturing relationships (for a review, see Newman et al., 2019), this study challenges this viewpoint by showing the potential benefits of SIU.

Second, we found empirical support for the mechanisms proposed by the DIRe model (Huxhold et al., 2022). Specifically, the results align with the capacities path that adopting less energy-consuming interaction resources positively affects investment opportunities, and thus the social network. Given this path, we find support for the assumption that SIU is indeed a way to communicate that requires less energy.

Third, population aging has resulted in healthcare policies favoring aging in place. Furthermore, due to cuts in formal care budgets, older adults must increasingly rely on informal caregivers to provide support and care in later life (Broese van Groenou & de Boer, 2016; Wiles et al., 2012). This stresses the importance of having sufficient possibilities to maintain a stable network. The results indicate that SIU can be one of these resources for older adults.

### Limitations

We must interpret our results considering some limitations. Participants answered the questions on SIU sequentially, first answering whether they used the internet, followed by whether and how often they used it to stay in contact with friends or family (our measure of SIU). However, the questions preceding these distinguish between a personal computer (i.e., laptop, desktop, or tablet) and a smartphone, asking how often participants use their smartphone to call, text, or *WhatsApp*. Our measure includes all online communication (and thus includes *WhatsApp* and texting). However, it is possible that participants may have excluded their smartphone use in the SIU questions due to the preceding smartphone questions, thereby underestimating their SIU rates and the resulting associations.

Our statistical choice of hybrid models allowed us to estimate cross-sectional and longitudinal associations (Allison, 2009), simultaneously looking at changes within and between respondents. In choosing this model, we assumed that SIU causes network changes, for which we found support. Additionally, reversed effects might be present, such that older adults’ social networks may influence their SIU (Roberts & Dunbar, 2011). Alternative analyses, such as the random intercept cross-lagged panel models (Hamaker et al., 2015), allow the testing of different causal pathways simultaneously. However, cross-lagged models produce biased results in the presence of stable confounder effects, especially when unmeasured (Lüdtke & Robitzsch, 2021). Because it is likely that many variables affect both internet use and network characteristics, we prioritized accounting for this heterogeneity.

## Conclusion

Our findings shed light on the potential of SIU in facilitating network maintenance in later life, which is complicated by the decreasing availability of energy due to health declines. We conclude that SIU can support older adults by offering a less energy-consuming means of communication. Our results show that more frequent SIU was associated with more continued and new ties, demonstrating the value of SIU in both maintaining and expanding the network.

## Supplementary Material

gbaf014_suppl_Supplementary_Materials

## Data Availability

Data from the Longitudinal Aging Study Amsterdam (LASA) are available for use for specific research questions, provided that an agreement is made up. Research proposals should be submitted to the LASA Steering Group, using a standard analysis proposal form that can be obtained from the LASA website: www.lasa-vu.nl. Files with data published in this publication are freely available for replication purposes and can be obtained using the same analysis proposal form. The LASA Steering Group will review all requests for data to ensure that proposals for the use of LASA data do not violate privacy regulations and are in keeping with informed consent that is provided by all LASA participants. The study was not preregistered.
