# Supplementary material for: Social Connectedness in Older Adults: The Potential of Social Internet Use to Maintain a Strong and Stable Personal Network
Source: J Gerontol B Psychol Sci Soc Sci. 2025 Jan 25;80(4):gbaf014. doi: 10.1093/geronb/gbaf014 (PMC11949425; doi:10.1093/geronb/gbaf014)
Supplement: gbaf014_suppl_Supplementary_Materials [file gbaf014_suppl_supplementary_materials.docx]

***The Journals of Gerontology, Series B: Psychological Sciences and Social Sciences* Supplementary Material: Janssen et al. Social connectedness in older adults: The potential of social internet use to maintain a strong and stable personal network.**

**Supplementary Table 1.** *Comparison of Various Cluster-Corrected Hybrid Regression Models of Contact Frequency on Social Internet Use*

**Supplementary Table 2.** *Comparison of Various Cluster-Corrected Linear Regression Models of Continued Ties on Social Internet Use*

**Supplementary Table 3.** *Comparison of Various Cluster-Corrected Linear Regression Models of Gained Ties on Social Internet Use*

**Supplementary Table 4.** *Comparison of Various Cluster-Corrected Linear Regression Models of Lost Ties on Social Internet Use*

# Supplementary Table 5. *Comparison of Various Cluster-Corrected Linear Regression Models*

| **Supplementary Table 1**  *Comparison of Various Cluster-Corrected Hybrid Regression Models of Contact Frequency on Social Internet Use* | | | | | | | |
| --- | --- | --- | --- | --- | --- | --- | --- |
|  | | Linear | | Ordinal | | Ordinal social internet use | |
| Variable | | *b* | *SE* | *b* | *SE* | *b* | *SE* |
| Cross-sectional associations | |  |  |  |  |  |  |
|  | Social internet use | –0.003 | 0.006 | –0.000 | 0.000 | – | – |
|  | Social internet use (ordinal) | – | – | – | – | –1.720^***^ | 0.445 |
|  | Age | –0.702^***^ | 0.096 | –0.020^***^ | 0.003 | –0.824^***^ | 0.099 |
|  | Network size | –1.031^***^ | 0.066 | –0.018^***^ | 0.002 | –1.000^***^ | 0.066 |
|  | Hours of work | 0.520^***^ | 0.065 | 0.011^***^ | 0.002 | 0.511^***^ | 0.065 |
|  | Health | 3.724^***^ | 0.803 | 0.094^***^ | 0.022 | 3.603^***^ | 0.806 |
|  | Co-residing partner^a^ | 5.809^***^ | 1.703 | 0.136^**^ | 0.046 | 5.899^***^ | 1.694 |
|  | Partner outside household^a^ | –3.432 | 3.827 | –0.119 | 0.103 | –2.617 | 3.796 |
| Longitudinal associations | |  |  |  |  |  |  |
|  | Social internet use | 0.003 | 0.003 | –0.000 | 0.000 |  |  |
|  | Social internet use (ordinal) |  |  |  |  | 0.252 | 0.357 |
|  | Age | –1.411^***^ | 0.114 | –0.078^***^ | 0.004 | –1.406^***^ | 0.114 |
|  | Network size | 0.867^***^ | 0.060 | 0.074^***^ | 0.002 | 0.866^***^ | 0.060 |
|  | Hours of work | 0.475^***^ | 0.067 | 0.016^***^ | 0.002 | 0.474^***^ | 0.067 |
|  | Health | 1.336^*^ | 0.655 | 0.080^***^ | 0.020 | 1.342^*^ | 0.654 |
|  | Co-residing partner^a^ | 0.292 | 2.090 | –0.215^**^ | 0.071 | 0.312 | 2.091 |
|  | Partner outside household^a^ | 0.651 | 2.921 | –0.188 | 0.106 | 0.663 | 2.922 |
| Time-invariant associations | |  |  |  |  |  |  |
|  | Female | –0.177 | 1.341 | 0.092^*^ | 0.038 | 0.035 | 1.333 |
|  | Years of education attained | –2.058^***^ | 0.192 | –0.045^***^ | 0.005 | –1.826^***^ | 0.189 |
| *Note*: SE = Standard error. 138,315 observations of 58,852 relationships clustered in 2,264 respondents.  ^a^ Reference category: no partner.  *** *p* < .05, ** *p* < .01, *** *p* <.001. | | | | | | | |

| **Supplementary Table 2**  *Comparison of Various Cluster-Corrected Linear Regression Models of Continued Ties on Social Internet Use* | | | | | | | | |
| --- | --- | --- | --- | --- | --- | --- | --- | --- |
| Variable | Linear | | Poisson | | Negative binomial | | Ordinal social  internet use | |
|  | *b* | *SE* | *b* | *SE* | *b* | *SE* | *b* | *SE* |
| Baseline social internet use | 0.002^**^ | 0.001 | 0.000^***^ | 0.000 | 0.000^***^ | 0.000 | – | – |
| Change in social internet use | 0.003^***^ | 0.001 | 0.000^***^ | 0.000 | 0.000^***^ | 0.000 | – | – |
| Baseline social internet use (ordinal) | – | – | – | – | – | – | 0.203^***^ | 0.060 |
| Change in social internet use (ordinal) | – | – | – | – | – | – | 0.248^***^ | 0.057 |
| Baseline age | –0.030^*^ | 0.014 | –0.002 | 0.002 | –0.001 | 0.001 | –0.025 | 0.014 |
| Change in age | 0.350^*^ | 0.151 | 0.033^*^ | 0.016 | 0.018 | 0.015 | 0.356^*^ | 0.151 |
| Baseline health | –0.270^*^ | 0.128 | –0.035^**^ | 0.013 | –0.031^*^ | 0.012 | –0.259^*^ | 0.129 |
| Change in health | –0.110 | 0.106 | –0.014 | 0.011 | –0.011 | 0.010 | –0.105 | 0.106 |
| Network size at T1 | 0.429^***^ | 0.015 | 0.029^***^ | 0.001 | 0.032^***^ | 0.001 | 0.428^***^ | 0.015 |
| Hours of work at T1 | –0.009 | 0.008 | –0.000 | 0.001 | –0.000 | 0.001 | –0.008 | 0.008 |
| Co-residing partner at T1^a^ | 1.700^***^ | 0.255 | 0.210^***^ | 0.029 | 0.211^***^ | 0.027 | 1.678^***^ | 0.255 |
| Partner outside household at T1^a^ | 0.284 | 0.433 | 0.023 | 0.054 | 0.018 | 0.054 | 0.288 | 0.429 |
| Female | 1.002^***^ | 0.221 | 0.115^***^ | 0.023 | 0.114^***^ | 0.021 | 1.024^***^ | 0.220 |
| Years of education attained | 0.069^*^ | 0.030 | 0.007^*^ | 0.003 | 0.007^*^ | 0.003 | 0.063^*^ | 0.031 |
| *Note:* SE = Standard error. 2,884 observations clustered in 1,624 respondents. In these analyses, the three waves have been restructured into two sets of two subsequent waves. ‘Baseline’ has the values of T1 and T2 for the two waves respectively, while ‘Change in’ has the values T2-T1 and T3-T2, respectively.  ^a^ Reference category: no partner.  *** *p* < .05, ** *p* < .01, *** *p* <.001. | | | | | | | | |

| **Supplementary Table 3**  *Comparison of Various Cluster-Corrected Linear Regression Models of Gained Ties on Social Internet Use* | | | | | | | | | | | |
| --- | --- | --- | --- | --- | --- | --- | --- | --- | --- | --- | --- |
|  | Linear | |  | Poisson | |  | Negative binomial | |  | Ordinal social  internet use | |
| Variable | *b* | *SE* |  | *b* | *SE* |  | *b* | *SE* |  | *b* | *SE* |
| Baseline social internet use | 0.006^***^ | 0.001 |  | 0.001^***^ | 0.000 |  | 0.001^***^ | 0.000 |  | – | – |
| Change in social internet use | 0.003^***^ | 0.001 |  | 0.001^***^ | 0.000 |  | 0.001^***^ | 0.000 |  | – | – |
| Baseline social internet use (ordinal) | – | – |  | – | – |  | – | – |  | 0.383^***^ | 0.058 |
| Change in social internet use (ordinal) | – | – |  | – | – |  | – | – |  | 0.306^***^ | 0.066 |
| Baseline age | 0.188^***^ | 0.014 |  | 0.040^***^ | 0.003 |  | 0.054^***^ | 0.004 |  | 0.194^***^ | 0.014 |
| Change in age | 1.741^***^ | 0.233 |  | 0.363^***^ | 0.044 |  | 0.381^***^ | 0.045 |  | 1.739^***^ | 0.233 |
| Baseline health | –0.217^*^ | 0.109 |  | –0.072^**^ | 0.025 |  | –0.090^***^ | 0.026 |  | –0.194 | 0.110 |
| Change in health | 0.036 | 0.131 |  | –0.001 | 0.029 |  | –0.016 | 0.032 |  | 0.044 | 0.131 |
| Network size at T1 | 0.088^***^ | 0.010 |  | 0.017^***^ | 0.002 |  | 0.019^***^ | 0.002 |  | 0.088^***^ | 0.010 |
| Hours of work at T1 | 0.009 | 0.006 |  | 0.002 | 0.002 |  | 0.004^*^ | 0.002 |  | 0.010 | 0.006 |
| Co-residing partner at T1^a^ | 0.394 | 0.217 |  | 0.118^*^ | 0.049 |  | 0.108^*^ | 0.050 |  | 0.340 | 0.216 |
| Partner outside household at T1^a^ | 0.185 | 0.426 |  | 0.060 | 0.090 |  | 0.047 | 0.093 |  | 0.211 | 0.426 |
| Female | 0.061 | 0.198 |  | 0.042 | 0.043 |  | 0.014 | 0.043 |  | 0.109 | 0.197 |
| Years of education attained | 0.068^*^ | 0.029 |  | 0.013^*^ | 0.006 |  | 0.005 | 0.006 |  | 0.067^*^ | 0.029 |
| *Note:* SE = Standard error. 2,884 observations clustered in 1,624 respondents. In these analyses, the three waves have been restructured into two sets of two subsequent waves. ‘Baseline’ has the values of T1 and T2 for the two waves respectively, while ‘Change in’ has the values T2-T1 and T3-T2, respectively.  ^a^ Reference category: no partner.  *** *p* < .05, ** *p* < .01, *** *p* <.001. | | | | | | | | | | | |

| **Supplementary Table 4**  *Comparison of Various Cluster-Corrected Linear Regression Models of Lost Ties on Social Internet Use* | | | | | | | | | | | |
| --- | --- | --- | --- | --- | --- | --- | --- | --- | --- | --- | --- |
|  | Linear | |  | Poisson | |  | Negative binomial | |  | Ordinal social  internet use | |
| Variable | *b* | *SE* |  | *b* | *SE* |  | *b* | *SE* |  | *b* | *SE* |
| Baseline social internet use | 0.001 | 0.001 |  | 0.000^**^ | 0.000 |  | 0.000^**^ | 0.000 |  | – | – |
| Change in social internet use | –0.001 | 0.001 |  | –0.000 | 0.000 |  | –0.000 | 0.000 |  | – | – |
| Baseline social internet use (ordinal) | – | – |  | – | – |  | – | – |  | –0.007 | 0.047 |
| Change in social internet use (ordinal) | – | – |  | – | – |  | – | – |  | –0.164^*^ | 0.070 |
| Baseline age | 0.018 | 0.011 |  | 0.007^***^ | 0.002 |  | 0.004^*^ | 0.002 |  | 0.014 | 0.011 |
| Change in age | 0.105 | 0.229 |  | –0.052 | 0.032 |  | 0.009 | 0.032 |  | 0.089 | 0.230 |
| Baseline health | 0.147 | 0.094 |  | 0.011 | 0.014 |  | 0.012 | 0.014 |  | 0.148 | 0.094 |
| Change in health | 0.048 | 0.124 |  | –0.002 | 0.017 |  | 0.004 | 0.018 |  | 0.046 | 0.124 |
| Network size at T1 | 0.394^***^ | 0.010 |  | 0.043^***^ | 0.001 |  | 0.036^***^ | 0.001 |  | 0.395^***^ | 0.010 |
| Hours of work at T1 | 0.008 | 0.006 |  | 0.002^*^ | 0.001 |  | 0.002^*^ | 0.001 |  | 0.008 | 0.006 |
| Co-residing partner at T1^a^ | –0.818^***^ | 0.180 |  | –0.064^*^ | 0.026 |  | –0.062^*^ | 0.027 |  | –0.829^***^ | 0.180 |
| Partner outside household at T1^a^ | –0.225 | 0.339 |  | –0.042 | 0.049 |  | –0.033 | 0.051 |  | –0.208 | 0.340 |
| Female | –0.585^***^ | 0.160 |  | –0.041 | 0.023 |  | –0.036 | 0.024 |  | –0.574^***^ | 0.159 |
| Years of education attained | 0.021 | 0.023 |  | 0.004 | 0.003 |  | 0.004 | 0.003 |  | 0.031 | 0.024 |
| *Note:* SE = Standard error. 2,884 observations clustered in 1,624 respondents. In these analyses, the three waves have been restructured into two sets of two subsequent waves. ‘Baseline’ has the values of T1 and T2 for the two waves respectively, while ‘Change in’ has the values T2-T1 and T3-T2, respectively.  ^a^ Reference category: no partner.  *** *p* < .05, ** *p* < .01, *** *p* <.001. | | | | | | | | | | | |

| **Supplementary Table 5**  *Comparison of Various Cluster-Corrected Linear Regression Models of Network Size on Social Internet Use* | | | | | | | | | | | | |
| --- | --- | --- | --- | --- | --- | --- | --- | --- | --- | --- | --- | --- |
|  | | Linear | |  | Poisson | |  | Negative binomial | |  | Ordinal social  internet use | |
| Variable | | *b* | *SE* |  | *b* | *SE* |  | *b* | *SE* |  | *b* | *SE* |
| Cross-sectional associations | |  |  |  |  |  |  |  |  |  |  |  |
|  | Social internet use | 0.016^***^ | 0.002 |  | 0.001^***^ | 0.000 |  | 0.001^***^ | 0.000 |  | – | – |
|  | Social internet use (ordinal) | – | – |  | – | – |  | – | – |  | 1.108^***^ | 0.125 |
|  | Age | –0.048 | 0.027 |  | –0.002 | 0.002 |  | –0.002 | 0.002 |  | –0.032 | 0.028 |
|  | Hours of work | 0.042^*^ | 0.017 |  | 0.002^*^ | 0.001 |  | 0.003^*^ | 0.001 |  | 0.048^**^ | 0.017 |
|  | Health | –1.116^***^ | 0.239 |  | –0.071^***^ | 0.014 |  | –0.072^***^ | 0.014 |  | –1.035^***^ | 0.240 |
|  | Co-residing partner^a^ | 4.856^***^ | 0.485 |  | 0.316^***^ | 0.028 |  | 0.316^***^ | 0.028 |  | 4.650^***^ | 0.485 |
|  | Partner outside household^a^ | 0.557 | 1.125 |  | 0.051 | 0.065 |  | 0.049 | 0.065 |  | 0.519 | 1.125 |
| Longitudinal associations | |  |  |  |  |  |  |  |  |  |  |  |
|  | Social internet use | 0.002 | 0.001 |  | 0.000^*^ | 0.000 |  | 0.000 | 0.000 |  |  |  |
|  | Social internet use (ordinal) |  |  |  |  |  |  |  |  |  | 0.231^*^ | 0.104 |
|  | Age | –0.399^***^ | 0.040 |  | –0.022^***^ | 0.002 |  | –0.022^***^ | 0.002 |  | –0.404^***^ | 0.039 |
|  | Hours of work | 0.009 | 0.015 |  | 0.000 | 0.001 |  | 0.000 | 0.001 |  | 0.009 | 0.015 |
|  | Health | –0.202 | 0.204 |  | –0.011 | 0.008 |  | –0.007 | 0.011 |  | –0.196 | 0.204 |
|  | Co-residing partner^a^ | 0.570 | 0.662 |  | 0.033 | 0.025 |  | 0.036 | 0.035 |  | 0.582 | 0.662 |
|  | Partner outside household^a^ | –0.406 | 0.831 |  | –0.020 | 0.033 |  | –0.008 | 0.046 |  | –0.411 | 0.830 |
| Time-invariant associations | |  |  |  |  |  |  |  |  |  |  |  |
|  | Female | 3.855^***^ | 0.412 |  | 0.251^***^ | 0.024 |  | 0.250^***^ | 0.023 |  | 3.949^***^ | 0.411 |
|  | Years of education attained | 0.149^*^ | 0.060 |  | 0.009^*^ | 0.003 |  | 0.009^**^ | 0.003 |  | 0.121^*^ | 0.061 |
| *Note:* SE = Standard error. 5,167 observations, clustered in 2,264 respondents.  ^a^ Reference category: no partner.  *** *p* < .05, ** *p* < .01, *** *p* <.001. | | | | | | | | | | | | |
